# Design of a Wearable Sensing System for Human Motion Monitoring in Physical Rehabilitation

**DOI:** 10.3390/s130607735

**Published:** 2013-06-17

**Authors:** Lara González-Villanueva, Stefano Cagnoni, Luca Ascari

**Affiliations:** 1 Henesis S.r.l., Viale dei Mille 108, 43125 Parma, Italy; E-Mail: luca.ascari@henesis.eu; 2 IBIS Lab, Department of Information Engineering, University of Parma, Parco Area delle Scienze 181/A, 43124 Parma, Italy; E-Mail: cagnoni@ce.unipr.it

**Keywords:** human motion monitoring, wireless body area networks, sensors, accelerometers, communication protocol, data synchronization, physical rehabilitation

## Abstract

Human motion monitoring and analysis can be an essential part of a wide spectrum of applications, including physical rehabilitation among other potential areas of interest. Creating non-invasive systems for monitoring patients while performing rehabilitation exercises, to provide them with an objective feedback, is one of the current challenges. In this paper we present a wearable multi-sensor system for human motion monitoring, which has been developed for use in rehabilitation. It is composed of a number of small modules that embed high-precision accelerometers and wireless communications to transmit the information related to the body motion to an acquisition device. The results of a set of experiments we made to assess its performance in real-world setups demonstrate its usefulness in human motion acquisition and tracking, as required, for example, in activity recognition, physical/athletic performance evaluation and rehabilitation.

## 1. Introduction

After suffering from a serious injury, illness or surgery, a patient typically must undergo a long and critical physical rehabilitation program to recover her/his former strength, mobility and fitness. Procedures for monitoring patients' movements are widely used in this context and are mainly aimed at identifying and maximizing life quality and movement potential. Many rehabilitation centers rely on classical treatments based on physiotherapy, which requires trained specialists and their precious experience. Sometimes, these treatments lack standardized and objective information that would be necessary for a proper evaluation of patients' performances. Along with the increased number of patients who suffer from motor function disability, this is the main reason why, since the 1980s, human motion tracking for rehabilitation has been an active research topic [1].

Motion sensors technology makes it possible to accurately identify, track and analyze movement. The data that can be acquired using such devices support the diagnosis and the rehabilitation process [2] by allowing therapists to precisely assess the impact of clinical interventions on the patients' everyday life and recovery [3]. Among the many different sensors that can be used for monitoring patients during rehabilitation, MEMS (*Micro*-*Electro*-*Mechanical Systems*) inertial sensors have been shown to have great potentials. The progress of miniaturization and their decreasing cost make it possible to incorporate them in compact, non-obtrusive continuous monitoring devices easily attachable to the body [4], empowering the development of Wireless Body Area Networks (WBANs) [5]. In particular, accelerometers can provide reliable information as well as objective and quantitative measurements when placed on different parts of the body [6].

Although many publications describe effective body motion data collection systems that rely on various multi-modal sensors, most of the proposed hardware devices or tracking systems include sensor/peripheral interconnection cables, not always flexible, and other components that have to be worn [7–10]. Because of this, such systems are usually not so comfortably or easily wearable and hamper the patient's movements. This makes them unsuitable for certain types of activities, such as the training programs in sports rehabilitation.

Our research has been aimed at developing a WBAN system that is comfortable to wear, easy to use, apply and re-apply, as well as non-limiting for the body movements and acceptable to clinicians. Such a system has a wide range of applications in several fields. As a result, we propose a prototypical system composed by wearable sensors, which has the following features:

- *Wireless communications*: the IEEE 802.15.4 standard is used to transfer the raw data from the sensors to the receiver.
- *Correctness of data*: the system represents the real situation with high measurement accuracy.
- *Real-time operation*: a real-time analysis of sensors' data is performed to provide the patient with an immediate feedback.
- *Portability*: the system components have limited size and weight, while being robust and permitting good mobility. This is particularly important, especially in the case of home-based therapies.
- *Easy manipulation*: the sensors are easy to use and to apply and re-apply on the body using elastic bands.
- *Automation*: the system can collect and store the patient's motion data automatically.
- *Friendly Graphical User Interface (GUI)*: the system includes an intuitive and simple user interface that makes it easy to use and displays the graphs of the data that are being acquired in real time.

The remainder of this paper is organized as follows. Section 2 presents the system design, including its architecture, configuration, communication protocol and data-alignment algorithm. Section 3 describes the experiments that have been carried out to evaluate the system performance and discusses their results. Finally, Section 4 draws some conclusions and anticipates future research goals.

## 2. System Design

### 2.1. System Architecture

**Figure 1.**
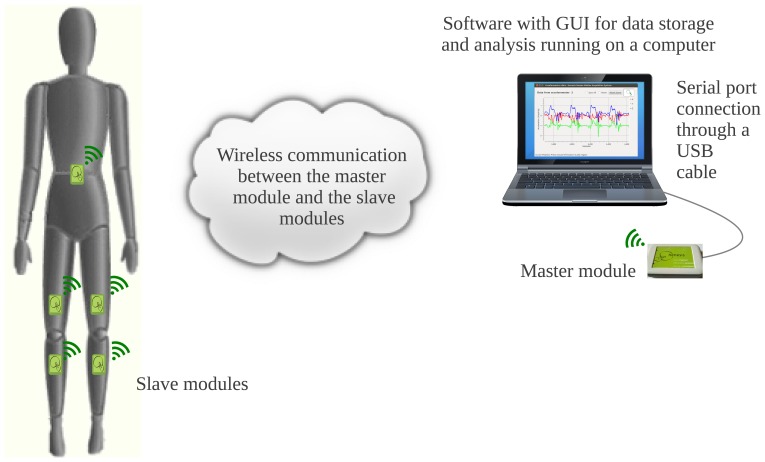
Proposed system architecture for human motion tracking and analysis.

Figure 1 shows the architecture proposed for our system. It includes six low-cost universal modules, one acting as a master and five as slaves. In the scheme shown in Figure 1, the slave modules are used to monitor the patient's knees, being placed one above and one below each knee, while the fifth is placed on the back waist of the patient, near the center of mass, to provide additional information about the patient's movement. Thin elastic bands are used to fix them to the body for easy wearability. Thanks to the system's flexibility, any other part of the body, e.g., the upper limbs, could be monitored just by changing the placement of the modules. The slave modules transmit their data wirelessly to the master module, whose main function is to keep the system synchronized while receiving the accelerometer data from the slaves and to store them into a computer or a monitoring station via a USB connection. A software application featuring a friendly GUI to control the sensing system has also been developed and can run under both Windows and Linux.

**Figure 2.**
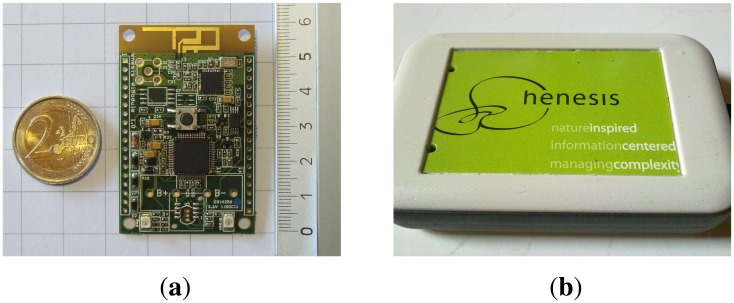
Details of the HenesisWiModule. (**a**) Size comparison; (**b**) Example of packaging.

The main component of the proposed architecture is the Henesis WiModule [11], shown in Figure 2. Its dimensions are 60 × 39 × 11 mm, which makes it a small wearable module, as shown in Figure 2(a). Figure 2(b) shows how the electronic board has been packaged for applications in rehabilitation, in order to easily attach it to an elastic band using a rear clip. It should be noticed that all modules are based on the same hardware, and may be programmed to act either as master or slave. The components of the Henesis WiModule that are more relevant to this work are a high-performance tri-axial accelerometer (LIS3LV02DQ from ST Microelectronics), a RF transceiver (MRF24J40 from Microchip) and an 8-bit PIC microcontroller (18F67J11 from the Microchip PIC18F87J11 family). Programming correctly the device and interconnecting these components appropriately is critical for the final performance of the system. The board also includes digital buses and analog lines, allowing for future addition of new external sensors.

### 2.2. System Configuration

The clock frequency of the system, for both the master and the slaves, has been set to the maximum allowed, *i.e.*, 32 MHz by using a 8 MHz oscillator and a Phase Lock Loop frequency multiplier configured with a value of 4×. A Transistor-Transistor Logic level serial port is used for transmitting the data from the master module to the computer; its transfer rate has been set to a high value, 460,800 bauds. The wireless communication section conforms the IEEE 802.15.4 standard [12]. It makes 16 channels available in the 2.4 GHz band, numbered from 11 to 26. Each of them has a bandwidth of 2 MHz and a channel separation of 5 MHz. Channel 26 was selected for the system's transmissions, as it is affected by fewer interferences than the others. The reported range of the wireless transmissions is up to 100 m outdoor, being in line-of-sight, and up to 20 m under typical indoor conditions. The accelerometer is capacitive since it guarantees higher stability than piezoelectric ones and it is therefore more suitable for measuring human motion [13]. According to the application's requirements and the clinicians' recommendations, 30 Hz is an adequate sampling rate. In fact, many of the products presented in [14] have this configuration. However, in order to have more data for posterior analysis, and considering the available bandwidth, we selected a higher frequency of 160 Hz. The scale has been set to ±6g as the results of the first tests demonstrated that the range ±2g was not wide enough for measuring motion in activities like running. This can be considered an adequate configuration according to [15], where it is shown that, for assessing daily physical activity, accelerometers should be able to measure accelerations up to ±6g. At the same time, this configuration extends the range reported by similar systems for human activity recognition [16] and health monitoring [17].

The structure of the data packet has been defined in compliance with the IEEE 802.15.4 standard. The latter defines four frame types—acknowledgement (ACK), data, beacon and MAC command frame, along with two modes of operation—beacon-enabled network or non-beacon-enabled network. In a beacon-enabled network, beacons are transmitted periodically by the Personal Area Network (PAN) coordinator and are mainly used to provide synchronization services between all the devices in the PAN. Instead, a network that is non-beacon-enabled does not transmit a beacon unless it receives a beacon request. In order to have more flexibility in the design, we have chosen to define a proprietary wireless networking protocol using the non-beacon-enabled network and implementing later the beacon internally.

**Figure 3.**
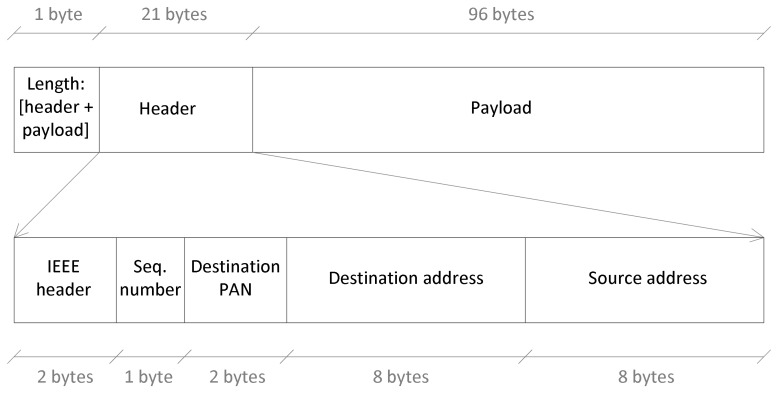
Structure of the data packet.

Figure 3 shows the structure of the data packet as provided to the transceiver of the module. The first byte indicates the packet length, including the header and the payload. The header length is 21 bytes: two bytes for the IEEE-compliant frame header that indicates, among other details, the type of packet; one byte for the sequence number; two bytes for the destination PAN; finally eight bytes for the destination address and eight more bytes for the source address.

The payload includes sixteen samples of the accelerometer to take full advantage of the packet size (filling the payload as much as possible reduces the protocol overhead). Each accelerometer sample is formed by 6 bytes, two for each of the three accelerometer axes: *x*, *y* and *z*. Therefore, the payload length is 96 bytes, and the packet that reaches the transceiver is composed by a total of 118 bytes. The transceiver adds one byte at the beginning that specifies the header length, and two additional bytes at the end specifying the Frame Check Sequence by using the Cyclic Redundancy Check. This final size of 121 bytes ensures the compliance with the IEEE 802.15.4 standard, which allows the payload of the MAC Protocol Data Unit to be variable, with the limitation that a complete MAC frame cannot exceed 127 bytes. As mentioned previously, the beacon is implemented internally by using a MAC command frame (specified in the IEEE frame header) with no payload, having a size of 23 bytes. On the other hand, the ACK frame, which is sent automatically by the transceiver if requested, has a size of 5 bytes.

### 2.3. Communication Protocol

**Figure 4.**
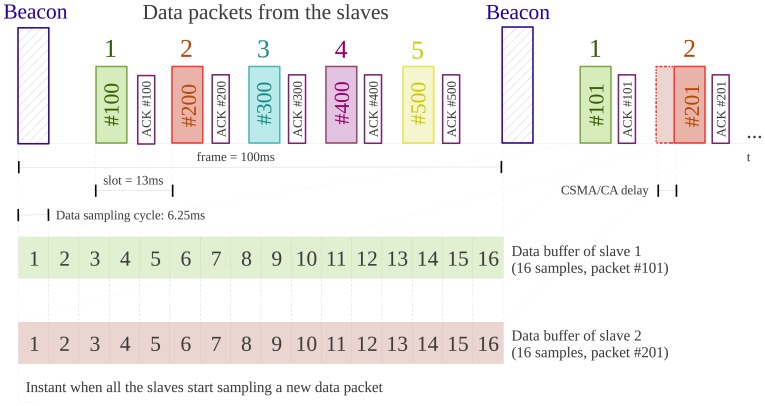
Communication protocol with data synchronization.

Taking the transmission medium into consideration, there would be high chances of losing packets or having collisions between them in the absence of a protocol that defines the system's behavior. Bearing in mind the target application, it is very important that the monitoring station receives all slave boards' data packets and in the correct order. The custom communication protocol that has been designed, which follows a Time Division Multiple Access (TDMA) approach and where data is sent upon request, is described below. For better clarity, the diagram of the protocol we implemented is shown in Figure 4.

The synchronization between the different modules is one of the relevant features of the system, whose structure, based on distributed sensors for data acquisition, brings up to a design where no implicit synchronization of the sensor nodes is present, as all nodes have independent clock sources [18]. A traditional synchronization scheme such as the *Network Time Protocol* (NTP) cannot be used because it is not suitable for sensor networks due to computing limitations and energy issues [19]. The protocol we have developed and we propose here is inspired by the *Reference-Broadcast Synchronization* (RBS), first published in [20]. As opposed to traditional protocols in which senders synchronize with receivers, in the RBS scheme, nodes send reference beacons to their neighbors, synchronizing a set of receivers with one another. Its fundamental property is that this reference broadcast does not contain an explicit timestamp; instead, receivers use its arrival time as a point of reference for comparing their clocks. Our protocol merges both strategies, using beacons for synchronizing implicitly the receivers among them and with the sender, the master module. It is therefore the master module the one in charge of sending the reference beacon to the slave modules. Unlike the RBS, in our proposal the receivers, *i.e.*, the slave modules, use the beacon arrival time, not to compare or readjust their clocks, but to start sampling a new data packet synchronously. As mentioned before, the beacon mode is implemented internally, so the master board sends periodically a beacon packet to the slaves to request their accelerometer data. The period is mainly determined by the number of data samples included in a data packet. Considering that there are sixteen samples in the packet and that the sampling frequency has been set to 160 Hz, the beacon will be sent approximately every 100 ms, depending on the jitter of the system, which will require a later study [21]. It must be pointed out that both the master and the slaves use the same transmission channel. Their transceivers have reception filters set, so that the master receives only data packets addressed to it, and the slaves receive only command or ACK packets from the master.

The protocol described in Figure 4 operates as follows: the master board sends a beacon packet, which is received by all the slaves at the same time. At that moment, each slave gets ready to send its data packet and starts acquiring a new one. If all the slaves started transmitting their data packets when they receive the beacon, collisions would occur. To avoid this situation, we have implemented a TDMA scheduling algorithm to share the transmission medium in time, so we assign each slave an ID number, depending on which each of them is allocated to a determined time slot to send its packets. During these time slots, the slaves send their data packets and wait till an ACK packet from the master is received. Having the ACK request configured permits to assure data reception and increases the robustness of the system. Another mechanism that has been implemented for this same reason is the Carrier Sense Multiple Access–Collision Avoidance (CSMA-CA), which avoids data collision when accessing the communication channel and improves communications reliability [22]. On the other side, when the master receives a data packet from a slave, it sends it the corresponding ACK packet and sends the data to the monitoring station through the serial port, so that the accelerometer samples are stored there. If the slave does not receive this ACK, it tries to send the same data packet for up to three trials, which is a native capability of the transceiver for packet retransmission. So, during the 100 ms interval between beacons (termed frame), each slave sends its data packet, waits for the ACK or retransmits the packet again if it has not been received. Anyway, to reach this stable situation, the system has to be synchronized first.

#### 2.3.1. Master Module Synchronization

When the system is first powered, the master starts an internal synchronization process to set the ideal timing interval for sending the beacon packets. Initially this was done directly every time it acquired 16 samples from its own on-board accelerometer. However, this was not very precise and introduced an undesired deviation of the period (jitter) in the system. To reduce it, we implemented a different strategy. During synchronization, the master module calculates how much time it takes on average to fill a data packet by using the accelerometer Interrupt Service Routine (ISR). When this process finishes, it uses the value it has obtained to program a timer and enters the normal operation mode. The ISR of this timer is responsible for sending the beacon packet periodically. This strategy reduces the jitter in this part of the system, as the hardware used to generate the time base for the timer, a crystal quartz, is very precise. In Table 1 we show the standard deviation of the jitter and its maximum value for both configurations. It can be observed that, when the programmed timer is used, the standard deviation is reduced by almost 50%.

#### 2.3.2. Slave Modules Synchronization and Specific Situations

On the slaves' side, when one receives a beacon packet for the first time, it also starts its own synchronization process. This requires that a certain number of consecutive beacon packets are received, so that the slave can estimate the average time between them. When the required number of beacon packets is received, the synchronization process finishes and the calculated average is used for setting a timer to start saving the data acquired from the accelerometer. The main goal of this approach is to synchronize the instant when all slaves start sampling data for a new packet. The accelerometer ISR is programmed with high priority to work independently, acquiring a sample every time one is ready, and keeping the mentioned timer in charge of copying the last sample acquired from the accelerometer into the data buffer. It is important to implement a buffering mechanism to avoid losing the samples that are to be sent with the next packet while transmitting the current one. To solve this problem, a double-buffer structure has been implemented, which makes it possible to use one buffer for sending a full packet while new data are being stored into the other buffer. To maintain data consistency it has also been specifically determined how to exchange the buffers. In this flow, the possible loss of beacon packets must also be checked. This is another task performed by the timer mentioned before. In case the beacon has been lost, or if there is an unexpectedly long delay, and the data buffer is full, this timer is responsible for exchanging the buffers and indicating that the packet is ready to be sent. If not, this is normally done by the transceiver routine when a beacon is received. It is necessary to differentiate between the situations where a beacon is lost, but the master is still awake (and, therefore, keeps receiving and saving data), or when it has been powered off. To take this into account, the slaves feature an embedded energy-saving mechanism that allows them to keep on sending their data packets only until a threshold number of lost beacons is reached. If this happens, they enter into the synchronization mode again, which requires that they receive a certain number of beacons before they can start sending new data to the master. If, instead, only one beacon has been lost, the slaves check that the beacon has not arrived when it should have, send directly the data packet that is ready and start sampling data for a new one. The possibility that the beacon arrives with a delay just after the data packet has been sent is also considered. In this case, and to maintain data synchronization, the samples that may have been acquired up to then are discarded, and the creation of a new packet is started. Regarding the situations in which a packet is retransmitted, this might happen because of two different events: the former occurs when the packet does not reach the master, the latter occurs when the master receives the data packet, but the ACK is lost. In both cases, which the slave cannot distinguish, it will retransmit the data packet.

### 2.4. Data Alignment

In the previous section we have explained how we synchronize the communication of all the slave modules and set the time when the sampling of a new data packet is started. The master module, just after receiving a data packet from a slave, transmits it to the monitoring station sequentially. In order to minimize the chances of transmission errors, or of an incorrect separation of a packet from the next one in the computer, start and stop delimiters are sent, too. Each of them is formed by 5 bytes and correspond to the sequences TXSTR and TXEND, respectively. The data contained in the packet is finally decoded and saved into the monitoring station. However, synchronization issues arise again when merging the data from different sensors. We designed a robust algorithm, described in Algorithm 1, in order to deal with situations in which beacon or data packets are lost, when a packet arrives with delay, or when a slave module is temporarily in synchronization mode. The main goal is to align the data from different accelerometers, to establish an exact correspondence between each of their packets. To facilitate the alignment task, every time a beacon is sent to the slave modules, the master device sends also a small packet to the computer through the serial port, which is detected thanks to the initial delimiter TXMST. This packet helps to divide the time into frames and to fuse the data of multiple sensors.



**Algorithm 1** Data alignment.
 Initialization; **while**
*not at end of the received data*
**do**  Read current packet;  **if**
*it is a beacon*
**then**   **if**
*previous frame is not completed*
**then**    Set a missing packet for each slave that missed it;   **end if**   Start a new frame;  **else**   Extract the source (slave ID) and the sequence number;   **if**
*received source* ! = *expected source*
**then**    Set a missing packet for the expected source;   **end if**   **if**
*received sequence number* ! = *expected one*
**then**    {At least one packet from this slave was lost before}    Cancel the missing packet if the current frame has already been filled;   **else**    **if**
*the frame has just been filled*
**then**     Cancel the missing packet as the right one has arrived late;    **end if**   **end if**   **if**
*there is a new packet but its current frame has already been filled with real data*
**then**    {The packet corresponds to a new frame}    **if**
*previous frame is not completed*
**then**     Set a missing packet for each slave that missed it;    **end if**    Start a new frame;   **end if**   Include the data packet in the frame and set it filled;   Update the expected source and sequence number;  **end if** **end while**


The combination of two different algorithms for time and data synchronization, named Multi-Data-Packaging and Slot-Data-Synchronization, is also used in the system-based design described in [23]. As their Slot-Data-Synchronization algorithm, our data alignment algorithm is implemented in the monitoring station. This choice allows us to reduce the computational load of the master module while keeping the time synchronization through the beacon reference as its main task.

## 3. Experimental Evaluation and Discussion

### 3.1. System Functionality

**Figure 5.**
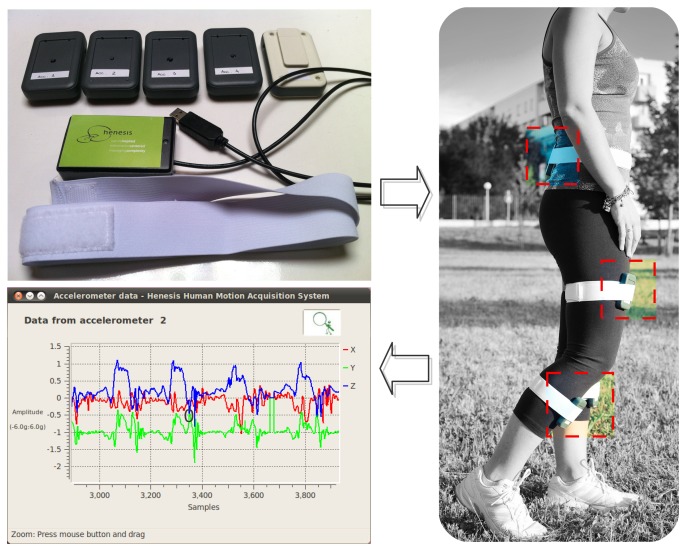
The proposed system, how to wear it and sample signals from one of the slave modules.

The prototypical hardware setup is shown in Figure 5. In the upper left image, there are the five slaves to be worn (as can be seen, in one of them there is a clip in the back part of the package that permits to attach the module to the elastic band shown below) and the master module, which is the one with the USB connection cable. The image on the right of Figure 5 shows a person wearing the slave modules as proposed in Figure 1. The lower left image shows the GUI with the graph of the waveforms corresponding to the three axes of the lower right leg accelerometer, acquired while the person was walking: one can see that there is a pattern in the movement performed, as expected. The figure demonstrates the use of the system in a real environment, the wireless connectivity between the slaves and the master module and how this kind of sensors makes it possible to capture body motion.

The size of the master module, once it has been packaged, is 90 × 55 × 22 mm, while the size of the slave modules is 84 × 52 × 16 mm. The package chosen for the slave modules allows to include light 3.6 V lithium ion rechargeable batteries, making them small self-powered and comfortably wearable modules. The weight of the slave modules, including battery, is 60 g. This solution overcomes the inconvenience of other systems, which require wearing a larger additional module on the waist containing the battery as well as several wires connected to the sensors, as described in [24]. This approach is also used in the commercial system described in [25]. The Xbus Master is a portable device, worn on the waist, which connects up to 10 inertial sensors and supplies power to them. Its size is 110 × 150 × 40 mm and its weight is 330 g, including batteries. On the other hand, this scheme allows the sensors to be smaller (38 × 53 × 21 mm) and lighter (30 g).

**Figure 6.**
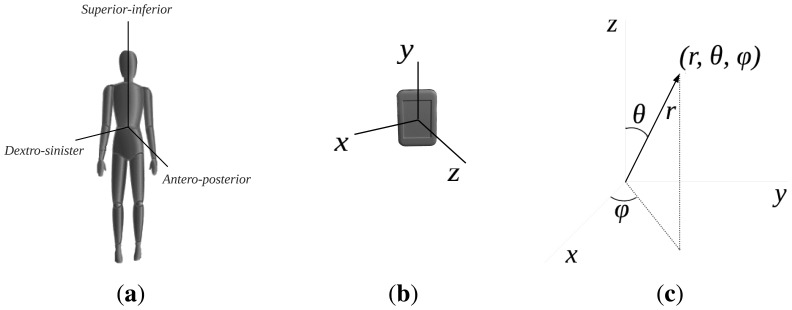
Coordinate reference system. (**a**) Human body reference axes; (**b**) Sensor coordinate system; (**c**) Cartesian and spherical coordinate systems.

To calibrate the system, the user needs to pose for a few seconds in four different pre-established postures. Considering how the accelerometer is mounted on the board (see the coordinate reference system in Figure 6), the expected reference values of every axis and accelerometer in those postures are saved in the system. After the acquisition, the results are compared with the expected ones, which allows one to determine the actual position of the accelerometers being worn and to correct their position, if necessary, before starting the exercises.

### 3.2. Communication Performance

The assessment of the communication performance takes into account four different aspects: data synchronization, data loss, jitter measurements and battery life.

#### 3.2.1. Data Synchronization

**Figure 7.**
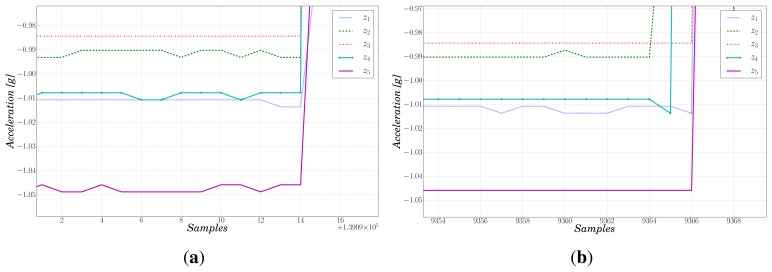
Zoom of the data for the synchronization test. (**a**) Best case: perfect synchronization; (**b**) Worst case: de-synchronization of two data samples.

As pointed out before, synchronization is a key aspect of the proposed system for later data analysis. To assess it, the so called “wooden bar experiment” was performed. The slave boards were fixed next to each other on a wooden bar with the purpose of assuring rigid mechanical connection. Afterwards, we hit the bar with a hammer with the aim to simulate a *δ* impulse. The main goal was to detect the start of the vibration produced by the hammer and check that it was consistently sensed by all modules. Figure 7(a) shows the accelerations along the *Z* axis of the five slaves, for the sake of clarity. We see that the beginning of the impulse is sampled simultaneously by all the accelerometers. The maximum de-synchronization among the modules corresponds to two data samples, *i.e.*, 12.5 ms, as shown in Figure 7(b). This is expected since the accelerometer sampling process relies on its own internal clock, which cannot be synchronized with the board's clock signal. This result represents an improvement with respect to the maximum error for data synchronization of a similar system made up of three sensors, proposed in [23], which was 24 ms.

#### 3.2.2. Data Loss

Considering the target application and the kind and duration of the exercises that are performed in a rehabilitation center, the experiments carried out for data acquisition to assess data loss and jitter had a duration of 15 minutes, which corresponds to the transmission of approximately 100,000 packets, considering beacon, ACK and data packets.

Table 2 shows the performance related to data loss (average, median and standard deviation) in two different environments, both considering ideal conditions in the lab (15 experiments) and real experiments while wearing the sensors (48 acquisitions from 10 different people). Although the number of lost packets increases in free-living environments, the high sampling frequency used permits to interpolate the missing information in the monitoring station without any noticeable degradation in the signal.

#### 3.2.3. Jitter Measurements

To validate the system, we also considered the time jitter of the beacon and data packets, calculated by using a wireless network analyzer in the experiments performed in the lab-controlled environment. The measurements are shown in Table 3. The average jitter is zero, which means that, in general, the packets are sent when expected. *jitter_σ_* represents the deviation from the expected value of the timing between the packets, while *jitter*_max_ corresponds to the maximum jitter. Both data help to infer the minimum possible duration of a time slot and, hence, the maximum number of slaves in the system. Allowing for a wide safety margin (the maximum jitter plus two times the standard deviation), that is having a time slot of approximately 13 ms, sets the maximum number of slaves that can be included in the system to five, as proposed. We can also see that the jitter is minimal in the case of the master, which validates the choice made for its configuration when sending the beacon packet. It should be mentioned that using the CSMA-CA mechanism slightly increases the jitter on the slaves, as explained in [26], while, on the other hand, it reduces data loss significantly.

#### 3.2.4. Battery Life

We also tested the battery life of the 3.6 V lithium ion rechargeable batteries used in the slave modules. Experiments have shown that they permit up to 12 hours of continuous operation, which is adequate for daily monitoring, although we aim to improve this aspect in the future.

### 3.3. Data Analysis

We present two different examples of data analysis, the former related to activity classification using raw data, and the latter focused on the extraction of relevant features for exercise assessment.

#### 3.3.1. Activity Classification

The aim of the first experiment was to demonstrate that the data obtained from the proposed system were meaningful. One person was asked to wear the slave modules as shown in Figure 5 and perform three different activities for 45 minutes: standing, sitting and walking. The acquired data was then separated into a training and a test set (60% and 40%, respectively). Afterwards, different classifiers were trained with the corresponding training set. We chose Weka [27], a collection of machine learning algorithms for data mining tasks, for the analysis of the data. The supervised methods selected for validating the system are: *J48*, a decision tree classifier [28], *SMO* (Sequential Minimal Optimization), a non-probabilistic linear binary classifier [29], and a probabilistic classifier, *Naive Bayes* [30]. The information obtained, without being pre-processed, was considered as a whole, *i.e.*, the classifiers were fed with all the 15 channels, 3 axes per each of the 5 accelerometers, at the same time. The results on the test set (over a total of 52,616 instances) are shown in Table 4. They demonstrate that the data obtained by the different sensors are meaningful and can be effectively used for activity classification.

#### 3.3.2. Pose Recognition and Exercise Assessment

**Figure 8.**
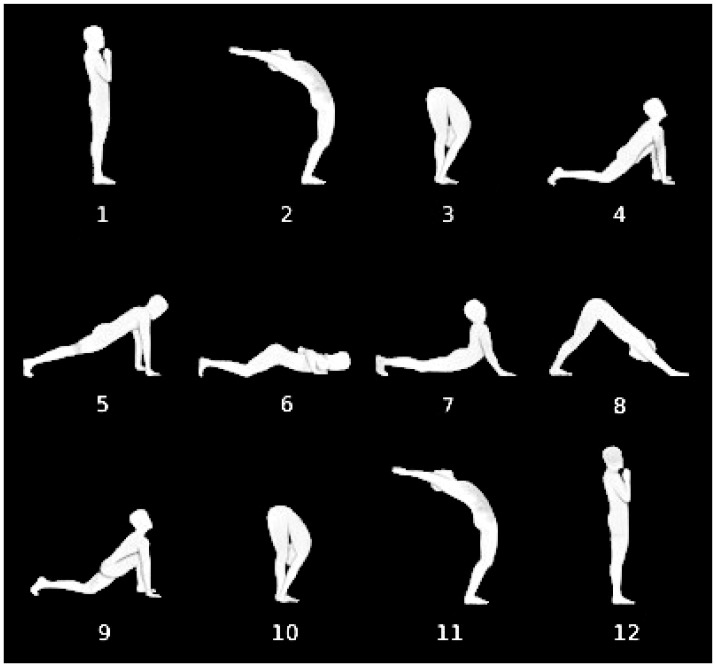
The twelve poses of the Sun Salutation sequence.

Yoga postures help to increase the tone of weak muscles and the alignment of the spinal column [31], which justifies its use in physical therapies, as in [32], where the effects of practicing yoga on motor variability, *i.e.*, strength, steadiness and balance, are assessed. It is important to perform the exercises at a slow pace with smooth, steady coordinated movements and having full control at every stage. Studying the pace of the movement of a subject is particularly relevant in the case of Parkinson's disease patients [33,34]. With the aim to validate the proposed system for application to this field, we collected the acceleration signals of a set of 10 healthy participants, who voluntarily took part in the evaluation, to assess the rhythm of the execution of the yoga Sun Salutation exercise, which is a flowing sequence of poses (see Figure 8). The motivation for choosing this exercise is that it includes a series of movements that involve various parts of the body, including both inferior and superior limbs, stretching nearly every part of it.

The subjects involved in the experiments were 7 men and 3 women with physical characteristics specified in Table 5. We asked each subject to perform the Sun Salutation exercise 4 times, producing a total of 4 datasets per subject.

In order to acquire meaningful data to model the movement appropriately, we took into consideration the postures assumed during the Sun Salutation. The sensors were placed as follows:

- Sensor 1 on the right forearm, above the hand.
- Sensor 2 on the left forearm, above the hand.
- Sensor 3 on the back waist, close to the center of mass of the body.
- Sensor 4 on the lower part of the right calf, over the Achilles tendon.
- Sensor 5 on the lower part of the left calf, over the Achilles tendon.

During the Sun Salutation, the movements of the body mainly occur along the anteroposterior and the superior-inferior axes, as shown in Figure 6(a). Taking into account how the exercises are performed in space, we realized that the movements could be modeled by considering the angle of the sensors with respect to the vertical axis as a relevant feature. Analyzing the data within this reference frame allowed us to reduce their dimensionality. Therefore, we decided to change from the Cartesian to the spherical coordinate system (see Figure 6(c)), obtaining the vector representation of each accelerometer and then extracting the angle, *θ*, with respect to the vertical axis, *Z*. This angle can be shown to be a relevant attribute with a resolution adequate for describing the human body movements while performing this sequence of poses. To recognize more accurately the transitions between the different poses of the exercise, five additional signals were considered, corresponding to the derivatives of the angles: $\left(\overset{d\theta_{1}}{\;}/\underset{dt}{\;},\overset{d\theta_{2}}{\;}/\underset{dt}{\;},\overset{d\theta_{3}}{\;}/\underset{dt}{\;},\overset{d\theta_{4}}{\;}/\underset{dt}{\;},\overset{d\theta_{5}}{\;}/\underset{dt}{\;},\right).$

For this specific application, we defined a custom calibration method in order to align the axes of the accelerometers to the same coordinate reference system, previously shown in Figure 6. At the beginning of the exercise, once the sensors are worn, the user must stay in a pre-defined position, just standing with the arms in a relaxed position along the body and the legs aligned, for a few seconds. In our convention, the Z axis is aligned with the gravity and is positive in the upper half-plane. Computing the corresponding rotation matrix and applying it to the data acquired, in this static position, all the accelerometers' vectors point to the floor, due to the gravity, with *θ* = 180°.

**Figure 9.**
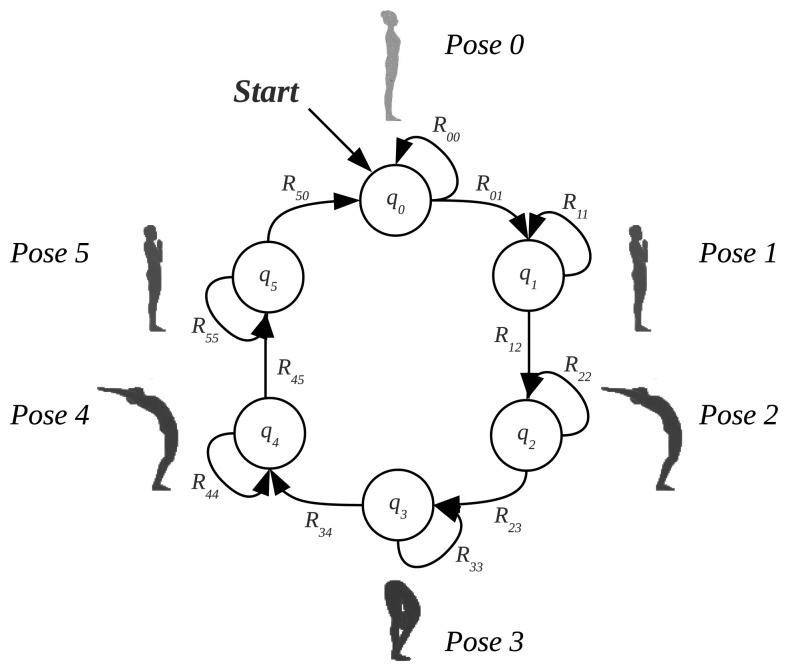
State diagram of the FFSM for the reduced cycle of the Sun Salutation.

The Fuzzy Finite State Machines (FFSMs) have demonstrated to be a suitable tool for modeling signals that evolve in time following a quasi-periodic repetitive pattern [35]. Analyzing the mentioned features by means of the FFSM represented in Figure 9 [36] we were able to recognize the poses represented next to the states, which correspond, for the proof of concept, to a reduced Sun Salutation sequence.

**Figure 10.**
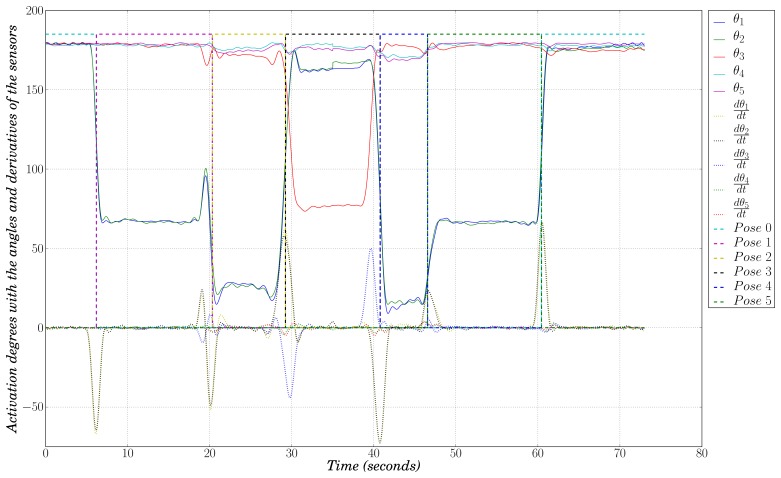
Pose recognition using the features extracted from the sensors.

The output of the FFSM contains the activation degree of every state at each instant in time, which means providing information related to pose recognition. As an example of the performance of our FFSM, Figure 10 plots the values, for one data acquisition session, of the angles and of their derivatives for each sensor (*θ_i_* and $\overset{d\theta_{i}}{\;}/\underset{dt}{\;}$, respectively) along with the activation degree of each state (*Pose j*, drawn in dashed lines). Take, for instance, the state *q*_0_, whose aim is to recognize *Pose* 0, which is the initial calibration pose. At the very beginning of the graph we see how the activation degree of the corresponding state is at high level while the other ones are at low level, which means that the first pose is being recognized by state *q*_0_ and therefore it is being identified as *Pose* 0. After a few seconds, the subject starts moving to the next pose (which can also be appreciated looking at the derivative signals), and during the transition, the activation degree of *Pose* 0 becomes low, while the activation degree of *Pose* 1 becomes high, recognizing the new pose. Figure 10 shows that our system is able to recognize adequately the evolution, through the six poses, of the selected sequence of movements.

From this analysis, we were able to give a preliminary feedback to the user, with information about the duration of the poses and the whole exercise. The duration refers to the amount of time during which each pose is recognized by the FFSM as the active pose. Table 6 shows the results obtained for each subject, reporting the average values and the corresponding standard deviation computed over the four datasets, for each of the sun salutation poses (state *q*_0_ is not included as it is related to the calibration pose). The last two columns summarize the average and standard deviation for the duration of all the poses and of the whole exercise. As can be seen, in general, the duration of state *q*_4_ is significantly shorter than that of the other states. This pose is considered the *most complex* of the sequence and, therefore, it is more difficult to maintain for a long period of time. The average duration of the poses and its standard deviation measure how uniformly the subject is performing the exercise, while the values referred to the whole exercise measure the homogeneity among different exercises performed by the same user. For example, Subject 2 performs the exercise at a smooth pace, but there are significant differences between the executions and even among the poses. Instead, Subject 6 performs the Sun Salutation with a more uniform duration of the poses and the various sequences present small differences in duration.

It is also interesting to mention that all the subjects were told to hold each position for 5 seconds while performing the exercise. The first two subjects had almost no external guidance, while the others were performing the exercise following an expert who was doing the same and another person modulating the pose execution and cadence. The statistics show that the external feedback improves exercise performance because of the unfamiliarity of the subjects with developing a pacing strategy, as suggested in [37].

## 4. Conclusions and Future Work

In this paper we have proposed a prototypical system for human motion monitoring, which can be used in rehabilitation therapy. The integration of five small wireless modules, worn by the subject, which can acquire accelerometer data at high frequency, synchronized by an external master device, makes the system ideal for patient monitoring since it is easily wearable and does not interfere with the movements. Furthermore, it provides significant flexibility, permitting to monitor different parts of the body with the same modules by just changing the placement of the elastic bands. The case study related to the Sun Salutation sequence requires the use of the five slave modules for the correct monitoring of all parts of the body, both the inferior and superior limbs, having a reference point next to the center of mass of the body. However, other exercises, such as monitoring a single arm during a rehabilitation therapy, could require fewer modules, being possible in this case to use the system in a simpler configuration without affecting its capabilities.

The resulting system operates in real time and in a wireless network, guaranteeing data correctness while being portable and easy to manipulate, which are crucial factors for the target application. In addition, it provides a software with a GUI for easy management of the sessions.

The successful working of the system has been demonstrated during the experiments carried out to assess the communication performance, which has been focused on data synchronization, data loss, jitter measurements and battery life. Furthermore, we have tested the system functionality analyzing the data acquired with it in the tasks of activity classification, pose recognition and exercise assessment using different techniques, from standard classifiers to Fuzzy Finite State Machines. We are able to provide a feedback to the subject about the pace of the movement, which is of particular importance in certain kinds of therapy. The results obtained show the potential of using our system in the field of human motion monitoring for rehabilitation.

However, the prototype can be further developed and improved. In the immediate future we aim to work on its miniaturization, by re-engineering the base module used in the architecture. This will allow to further reduce the size and weight of the wearable slaves. On the data analysis side, future work will also include both providing a linguistic description of the exercise performed, with information about symmetry, stability and rhythm, and analyzing the data using specific neuromorphic algorithms. The collaboration with a rehabilitation center will permit to define the data analysis tasks for each specific purpose and to complete the field validation of the system.

## Figures and Tables

**Table 1.** Standard deviation and maximum value of the beacon jitter with two different configurations.

**Configuration**	*jitter_σ_* (*μ*s)	*jitter*_max_ (*μ*s)
*Using accelerometer timing*	20.76	93.67
*Using programmed timer*	11.70	67.17

**Table 2.** Performance related to data loss.

**Environment**	**Statistic**	**Lost packets (%)**
**Lab-controlled**	*Average*	0.000
	*Median*	0.000
	*σ*	0.001

**Free-living**	*Average*	3.160
	*Median*	3.380
	σ	1.580

**Table 3.** Jitter measurements.

**Module**	**Statistic**	*jitter_σ_* (%)	*jitter*_max_ (%)
**Master**	*Average*	0.01	0.07
	*Median*	0.01	0.08
	*σ*	0.00	0.01

**Slaves**	*Average*	1.09	8.56
	*Median*	1.07	8.83
	*σ*	0.07	2.65

**Table 4.** Activity classification results on the test set.

***J48***		**Predicted Activity (%)**			**Total Accuracy**

		***Standing***	***Sitting***	***Walking***	
Actual activity	*Standing*	100.00	0.00	0.00	99.98%
	*Sitting*	0.00	100.00	0.00	
	*Walking*	0.09	0.00	99.91	



***SMO***		**Predicted Activity (%)**			**Total accuracy**

		***Standing***	***Sitting***	***Walking***	
Actual activity	*Standing*	100.00	0.00	0.00	99.89%
	*Sitting*	0.00	100.00	0.00	
	*Walking*	0.57	0.00	99.43	



***Naive Bayes***		**Predicted Activity (%)**			**Total Accuracy**

		***Standing***	***Sitting***	***Walking***	
Actual activity	*Standing*	98.77	0.00	1.23	99.49%

	*Sitting*	0.00	99.98	0.02	

	*Walking*	0.00	0.00	100.00	

**Table 5.** Subject characteristics (mean and, in parenthesis, standard deviation).

**Gender**	**Age (years)**		**Height (cm)**		**Weight (kg)**		**Body Mass Index (kg/m^2^)**	
Male	27.86	(2.12)	182.71	(8.54)	82.71	(12.87)	24.69	(2.66)
Female	28.67	(2.08)	162.33	(9.24)	53.33	(2.31)	20.30	(1.38)

**Table 6.** Analysis of poses and exercise duration (mean and, in parenthesis, standard deviation, in seconds).

**Subject**	**Duration of Each State**				**Total Summary**		

	*q*_1_	*q*_2_	*q*_3_	*q*_4_	*q*_5_	**Pose**	**Exercise**
**1**	13.32 (0.82)	8.57 (0.40)	11.91 (2.78)	6.93 (1.24)	13.07 (1.08)	**10.75 (2.94)**	**53.80 (3.17)**
**2**	11.25 (1.38)	8.94 (1.62)	11.38 (1.18)	7.81 (0.82)	14.68 (3.95)	**10.81 (3.06)**	**54.04 (6.98)**
**3**	7.86 (0.69)	7.85 (0.69)	10.03 (1.91)	0.84 (0.09)	12.83 (4.05)	**7.88 (4.46)**	**39.41 (3.55)**
**4**	7.71 (0.76)	11.16 (8.64)	6.06 (3.51)	2.88 (2.39)	7.11 (4.94)	**6.98 (5.11)**	**34.92 (3.31)**
**5**	9.43 (5.03)	7.06 (0.28)	8.69 (1.49)	1.92 (1.47)	8.75 (2.54)	**7.17 (3.69)**	**35.84 (1.25)**
**6**	6.41 (0.61)	6.32 (0.63)	6.68 (0.46)	5.01 (0.47)	5.52 (0.66)	**5.99 (0.82)**	**29.95 (0.83)**
**7**	6.84 (1.13)	6.94 (1.02)	7.19 (0.51)	2.43 (1.72)	8.33 (3.09)	**6.35 (2.59)**	**31.73 (3.44)**
**8**	6.61 (0.21)	6.26 (0.76)	8.16 (0.12)	1.93 (1.78)	9.72 (2.32)	**6.53 (2.94)**	**32.67 (1.56)**
**9**	6.52 (0.74)	5.91 (0.27)	7.89 (0.69)	6.71 (0.63)	6.43 (0.71)	**6.69 (0.88)**	**33.46 (2.77)**
**10**	12.80 (0.38)	0.29 (0.10)	7.42 (0.67)	6.25 (0.34)	5.47 (0.51)	**6.44 (4.13)**	**32.22 (1.08)**

## References

[1] Zhou H., Hu H. (2008). Human motion tracking for rehabilitation—A survey. Biomed. Signal Process. Control.

[2] Patel S., Park H., Bonato P., Chan L., Rodgers M. (2012). A review of wearable sensors and systems with application in rehabilitation. J. Neuro Eng. Rehabil..

[3] Bonato P. (2005). Advances in wearable technology and applications in physical medicine and rehabilitation. J. NeuroEng. Rehiabil..

[4] Moreno-Hagelsieb L., Tang X., Bulteel O., Overstraeten-SchloÖgel N.V., Andreé N., Dupuis P., Raskin J.P., Francis L., Flandre D., Fonteyne P. Miniaturized and Low Cost Innovative Detection Systems for Medical and Environmental Applications.

[5] Latré B., Braem B., Moerman I., Blondia C., Demeester P. (2011). A survey on wireless body area networks. Wirel. Netw..

[6] Yang J., Wang S., Chen N., Chen X., Shi P. Wearable Accelerometer Based Extendable Activity Recognition System.

[7] Guler M., Ertugrul S. Measuring and Transmitting Vital Body Signs Using MEMS Sensors.

[8] Zhou H., Hu H., Tao Y. (2006). Inertial measurements of upper limb motion. Med. Biol. Eng. Comput..

[9] Zhou H., Stone T., Hu H., Harris N. (2008). Use of multiple wearable inertial sensors in upper limb motion tracking. Med. Eng. Phys..

[10] Sung M., Marci C., Pentland A. (2005). Wearable feedback systems for rehabilitation. J. NeuroEng. Rehabil..

[11] Henesis WiModule. http://www.henesis.eu/prod-wimodule-eng.htm.

[12] LAN/MAN Standards Committee–IEEE Computer Society (2011). IEEE Standard for Local and Metropolitan Area Networks–Part 15.4: Low-Rate Wireless Personal Area Networks (LR-WPANs).

[13] Tao W., Liu T., Zheng R., Feng H. (2012). Gait analysis using wearable sensors. Sensors.

[14] Yang C.C., Hsu Y.L. (2010). A review of accelerometry-based wearable motion detectors for physical activity monitoring. Sensors.

[15] Bouten C., Koekkoek K., Verduin M., Kodde R., Janssen J. (1997). A triaxial accelerometer and portable data processing unit for the assessment of daily physical activity. IEEE Trans. Biomed. Eng..

[16] Ravi N., Nikhil D., Mysore P., Littman M.L. Activity Recognition from Accelerometer Data.

[17] Milenković A., Otto C., Jovanov E. (2006). Wireless sensor networks for personal health monitoring: Issues and an implementation. Comput. Commun..

[18] Ahmed S., Chen T. (2011). Minimizing the effect of sampling jitters in wireless sensor networks. IEEE Signal Process. Lett..

[19] Sivrikaya F., Yener B. (2004). Time synchronization in sensor networks: A survey. IEEE Netw..

[20] Elson J., Girod L., Estrin D. Fine-grained Network Time Synchronization Using Reference Broadcasts.

[21] Paavola M., Kemppainen J. Wireless Monitoring of a Steam Boiler-performance Measurements in Industrial Environment.

[22] Lee J.S. (2006). Performance evaluation of IEEE 802.15.4 for low-rate wireless personal area networks. IEEE Trans. Consum. Electron..

[23] Mo L., Liu S., Gao R., John D., Staudenmayer J., Freedson P. (2012). Wireless design of a multi-sensor system for physical activity monitoring. IEEE Trans. Biomed. Eng..

[24] Kunze K., Lukowicz P. Using Acceleration Signatures from Everyday Activities for On-body Device Location.

[25] XSens Xbus Kit: Measurement of human motion. http://www.xsens.com/en/general/xbus-kit.

[26] González-Villanueva L., Chiesi L., Mussi L. Wireless Human Motion Acquisition System for Rehabilitation Assessment.

[27] Hall M., Frank E., Holmes G., Pfahringer B., Reutemann P., Witten I.H. (2009). The WEKA data mining software: An update. SIGKDD Explor. Newslett..

[28] Quinlan J.R. (1993). C4.5: Programs for Machine Learning.

[29] Platt J. (1998). Fast Training of Support Vector Machines using Sequential Minimal Optimization.

[30] Mitchell T. (1997). Machine Learning.

[31] Nayak N.N., Shankar K. (2004). Yoga: A therapeutic approach. Phys. Med. Rehabil. Clin. N. Am..

[32] Hart C.E.F., Tracy B.L. (2008). Yoga as steadiness training: Effects on motor variability in young adults. J. Strength Cond. Res..

[33] Platz T., Brown R.G., Marsden C.D. (1998). Training improves the speed of aimed movements in Parkinson's disease. Brain.

[34] Majsak M.J., Kaminski T., Gentile A.M., Flanagan J.R. (1998). The reaching movements of patients with Parkinson's disease under self-determined maximal speed and visually cued conditions. Brain.

[35] Alvarez-Alvarez A., Trivino G., Cordón O. (2012). Human gait modeling using a genetic fuzzy finite state machine. IEEE Trans. Fuzzy Syst..

[36] González-Villanueva L., Alvarez-Alvarez A., Ascari L., Trivino G. Computational Model of Human Body Motion Performing a Complex Exercise by Means of a Fuzzy Finite State Machine.

[37] Carroll C.M., Dixon C.B. (2011). The effect of feedback on exercise performance in recreationally-active young adults. Keyst. J. Undergrad. Res..

